# Microanatomy of incremental growth lines in dental tissues in reindeer *Rangifer tarandus*

**DOI:** 10.1111/joa.14135

**Published:** 2024-09-05

**Authors:** Alan Boyde, Nicholas J. C. Tyler

**Affiliations:** ^1^ Dental Physical Sciences Unit, Institute of Dentistry Queen Mary University of London London UK; ^2^ Centre for Saami Studies UiT the Arctic University of Norway Tromsø Norway; ^3^ Department of Agricultural Sciences Lincoln University Christchurch New Zealand

**Keywords:** age determination, annulations, cementochronology, incremental lines, periodontology, *Rangifer tarandus platyrhynchus*, scanning electron microscopy

## Abstract

Counting growth layers in dentine and/or secondary cementum is widely used for age determination in wild mammals but the underlying seasonal changes in the structure and degree of mineralisation of dental tissue have not been well characterised. We embedded first (m1) and second (m2) mandibular permanent molar teeth from a 12‐year‐old female Svalbard reindeer (*Rangifer tarandus platyrhynchus*) in PolyMethylMethAcrylate (PMMA), prepared cut and polished surfaces coated with evaporated carbon and used 20 kV back‐scattered electron imaging in a scanning electron microscope (BSE‐SEM) to study aspects of dental tissue structure which depend on the degree of mineralisation at the micron and sub‐micron scale. BSE‐SEM revealed differences between the mineral content of growth layers (annulations) in the secondary cementum and the primary and secondary dentine, the latter, incidentally, still forming at death in m1. Wide bands of less well mineralised tissue formed in the cementum during active appositional phases. Thin, denser bands formed by maturation‐mineralisation of existing tissue when growth slowed in winter. This maturation mimics the processes seen in lamellar bone and articular cartilage. Counter to previous suggestions, there was evidence of substantial resorption and repair of the secondary cementum and of formation of dentine throughout life. Secondary dentine is layered by mineral content like cementum. In the crown, this was mainly tubular dentine with well‐marked interglobular dentine layers. In the lower pulp chamber and root, it was largely without tubules. Substantial non‐mineralised spaces found at the cement‐dentine junction in the root apical regions in m2 represent inclusions of the Hertwig's Epithelial Root Sheath (HERS) or the Epithelial Rests of Malassez (ERM) between the two tissues, a phenomenon which has previously only been identified in Muridae. The anatomical changes which result in the formation of the incremental lines (annulations) in dental tissues of reindeer, identified here for the first time at the micrometre level, are likely to be common across most if not all long‐lived species of mammals living in seasonal environments.

## Graphical abstract

The nature of the incremental lines in reindeer cementum was investigated using back‐scattered electron imaging in an SEM. Winter rest lines form when matrix deposition stops, but mineralisation continues by a process analogous to maturation in bone and the formation of multiple tidemark lines in articular calcified cartilage.

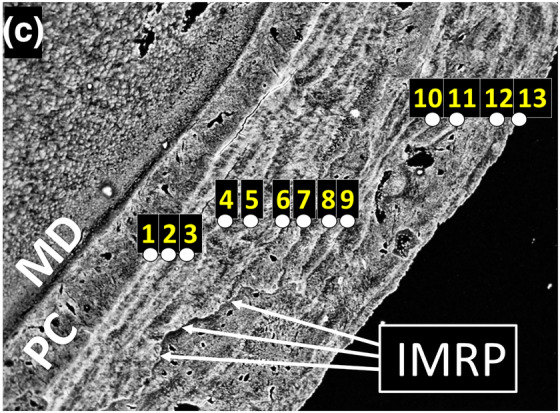

## 1 INTRODUCTION

The presence of growth layer lines in (or on) dental hard tissues is routinely exploited by zoologists and zooarchaeologists to determine age at death in individual wild mammals (Scheffer, 1950; Laws, 1952, 1953; Sergeant, 1959; Sergeant & Pimlott, 1959; Mansfield & Fisher, 1960; Low & Cowan, 1963; Mitchell, 1963, 1967; Ransom, 1966; Klevezal & Kleinenberg, 1967; Reimers & Nordby, 1968; Miller, 1974; Leader‐Williams, 1979; Grue & Jensen, 1979; Perrin & Myrick, 1980). This method rests on the assumption that the rate and/or the nature of deposition of tissue, and hence the formation of the lines, is influenced by, and leaves a permanent record of, the annual environmental cycle. The layering of cementum has thus been attributed to seasonal (i.e., once per annum) changes in food supply, nutritional quality of food and food intake, and hence in seasonal rates of growth and metabolism, as well as in annual features of life history including seasonal migration and reproduction (Cerrito et al., 2020; Klevezal & Kleinenberg, 1967; Lieberman, 1994; Reimers & Nordby, 1968), and the resulting lines are referred to as ‘annulations’ (sometimes ‘annuli’) for that reason. Counting growth layers in dental cementum to determine age at death routinely follows methodologies that were developed decades ago and which have altered little since (e.g., Miller, 1974; Sergeant & Pimlott, 1959; Veiberg et al., 2020). Commercial and government laboratories provide this service at relatively low cost and the results are implicitly relied on. Thirty years ago, however, Lieberman (1994) remarked that cementum increment analysis was a black‐box technique because the factors that cause bands have been poorly understood and this remains largely true today.

Cementum rather than dentine or enamel is chosen because it is laid down mainly at a periphery (i.e., on the ‘outside’ of the tooth) where the presence of a surface on which further growth can occur means that it may form throughout life. With the exception of very large animals, incremental lines in cementum can usually only be seen by microscopic examination of thin sections. Sections can be cut in different planes but, while it is far easier to cut transverse sections [TSs] than longitudinal sections [LSs], the latter make more sense for age estimation because they sample the entire developmental history of the tooth.

Age determination is commonly based on examination of cementum around the roots of permanent incisors, normally i1, which are easier to extract and handle than molars. The starting point or ‘zero‐line’ for counting annulations is the structure assumed to have been formed during the first winter of life (the first ‘winter’ or ‘rest line’; Reimers & Nordby, 1968) before the tooth erupted or even was fully formed. As a general principle, however, it is better to choose a tooth which contains, as a starting point, a layer for which the age can safely be assumed to be zero years. The only tooth in a diphyodont dentition which contains a neonatal line or in which, failing that, deposition of calcified tissue is known to start shortly after birth, is normally the first permanent molar (m1). In reindeer/caribou *Rangifer tarandus* (hereafter ‘*Rangifer*’) and red deer (*Cervus elaphus*) m1, the first permanent tooth to erupt, is fully functional at 3 to 5 and 4 to 6 months, respectively (Miller, 1974; Mitchell, 1963, 1967). Mitchell (ibid.) concluded that m1 is the best tooth for age determination based on counting cementum lines and that the best place to count lines in the red deer m1 was in what he called the ‘cement pad’ between the two roots.

Accurate age determination requires that layering happens in all the cementum irrespective of its level on the tooth. This, however, is not invariably the case in mammals: for instance, growth layering in secondary cementum in *Homo sapiens* only starts when a root is nearly or fully formed and therefore roughly halfway down the root. Another essential condition is that the layering of tissue leaves a permanent record. However, piecemeal resorption and repair of cementum is common, and counting is only legitimate where there are ‘good’ regions that have been left unmolested by osteoclasts.

Particular aspects of the anatomy of annulations remain unclear. While Lieberman (1994) stated that ‘Most researchers agree that bands of cementum which differ in their relative percentage of mineral would be optically distinct in both polarised and non‐polarised light, but differences in the relative mineralisation of bands have rarely been tested and there is little agreement about which bands are more mineralised’, Cool et al. (2002), however, concluded that cementum growth layers were not the result of changes in mineral density but relate, rather, to mineral orientation.

Few studies have attempted to define structural changes in growth layers at the micrometre scale (Bosshardt & Schroeder, 1990; Boyde, 1980; Perrin & Myrick, 1980; Schroeder, 1993). The purpose of the present study was to investigate the micro‐anatomical basis for their existence to substantiate the use of counting incremental lines for age‐determination. Thus, we employed back‐scattered electron imaging scanning electron microscopy [BSE‐SEM] to study variation in the concentration of minerals in calcified tissues in an m1 and an m2 from a Svalbard reindeer *Rangifer tarandus platyrhynchus*. For flat surfaces, BSE‐SEM gives information comparable to X‐ray projection microscopy or microradiography of plane‐parallel thin sections (e.g., Cool et al., 2002; Lieberman, 1994) but at a far better volumetric resolution (Boyde & Jones, 1983).

## 2 MATERIALS AND METHODS

Two molar teeth were obtained from an adult female Svalbard reindeer known to have died of natural causes in Adventdalen, Svalbard in the winter of 1978/1979 (Tyler et al., 2008). NT examined incisor sections (i1) from this animal, prepared by the method of Miller (1974) and counted 12 annulations. The outermost layer stained dark and was designated a ‘winter’ or ‘rest’ line consistent with the death of the animal in winter.

**FIGURE 1:**
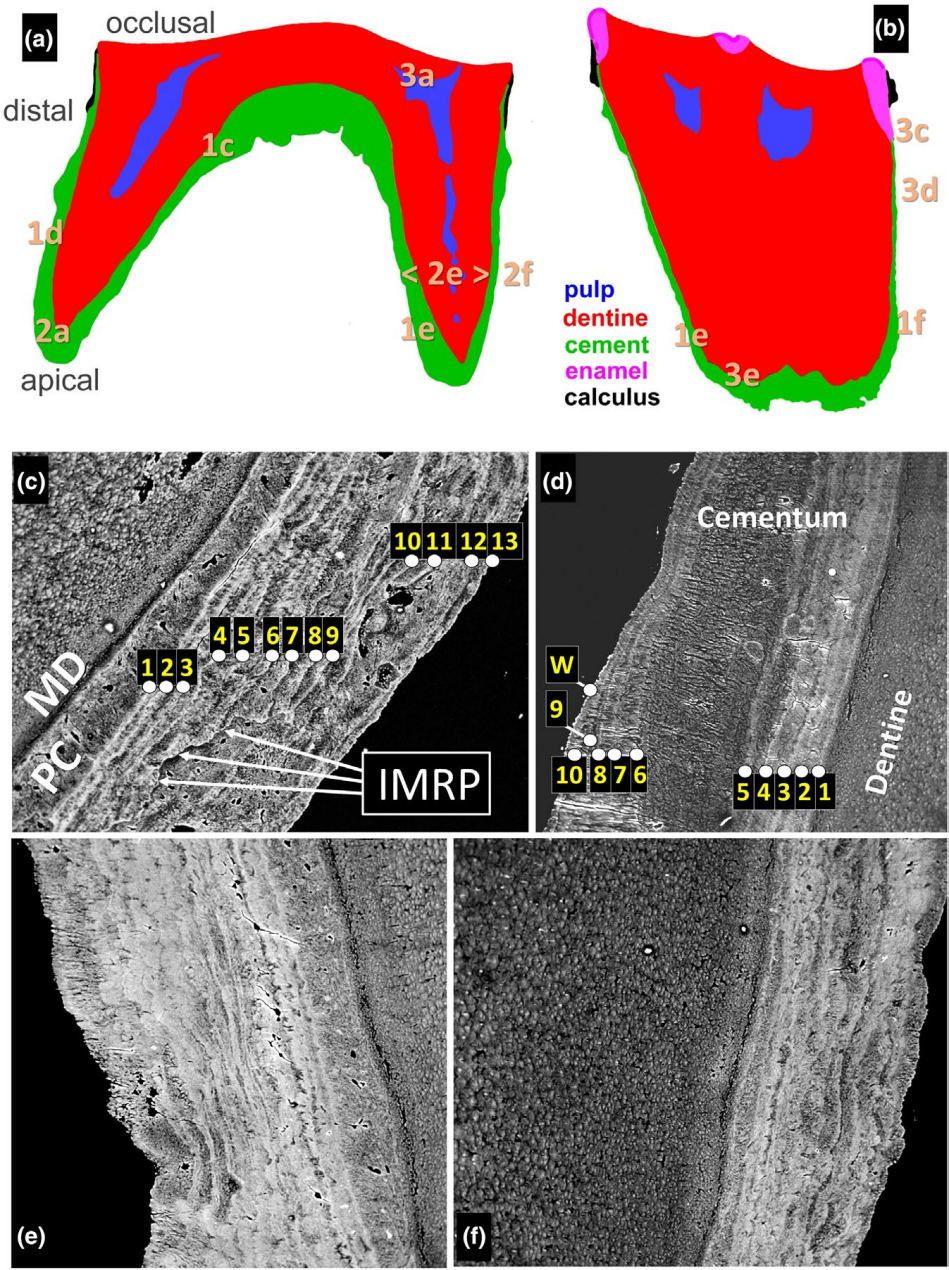
BSE‐SEM showing cementum growth layers (annulations) in m1 and m2. (a, b) Outlines showing the distribution of the dental tissues in the m1 (a) and m2 (b) samples and the locations of the fields shown in the following figures. Where different magnifications on the same centre are used, just the first figure part location is indicated. (c) Mesial side of distal root of m1. Annulations in the cementum. Each annulation appears as a broad dark (i.e., less well mineralised) band, representing rapid growth and mineralisation in summer, and a thin ‘winter’ or ‘rest’ line representing slowed rate of growth in winter. This animal was determined to have survived 12 winters (rest lines labelled 1–13) and therefore to have been aged 12 whole years) when she died during her 13th winter. MD Mantle dentine. PC Primary cementum. IMRP Inner margin of a large, deep resorption patch which, having ‘eaten through’ several annual increments, shows repair with cellular cementum. Field width (FW) 830 μm. (d) Distal side of distal root of m1 with a thick increment between annulations 5 and 6. NB: Only 11 annulations, including the lattermost winter rest line (W), are distinct in this section of the tooth. FW 415 μm. (e) Annulations in cementum on the lingual side of the mesial root of m2. FW 413 μm. (f) Annulations in cementum on the buccal side of the mesial root of m2. FW 450 μm.

The teeth were embedded in PMMA, cut longitudinally, polished and coated with carbon by evaporation (Boyde, 2019; Boyde & Jones, 1983). The first molar was sectioned mesio‐distally through the lingual cusps (Figure 1a); the second molar bucco‐lingually through the mesial cusps (Figure 1b). The blocks were examined in a Cambridge Instrument Co. Stereoscan Mark IV SEM at 20 kV. BSE images were recorded on 35 mm film and later scanned into digital format at 3200 dpi.

## 3 RESULTS

We describe aspects of cemental growth lines related to variation in the mineral concentration as characterised by BSE‐SEM and other details of the dental histology.

The distribution of the dental tissues is shown in Figure 1a,b. Enamel was present in m2 (Figure 1b) but not m1 (Figure 1a). Both teeth had calculus—calcified bacterial dental plaque—which was attached to cementum in m1 and enamel and cementum in m2 (Figure 1a,b). After enamel, calculus is the next most densely mineralised dental ‘tissue’: both are normally lost in any decalcification procedure.

**FIGURE 2:**
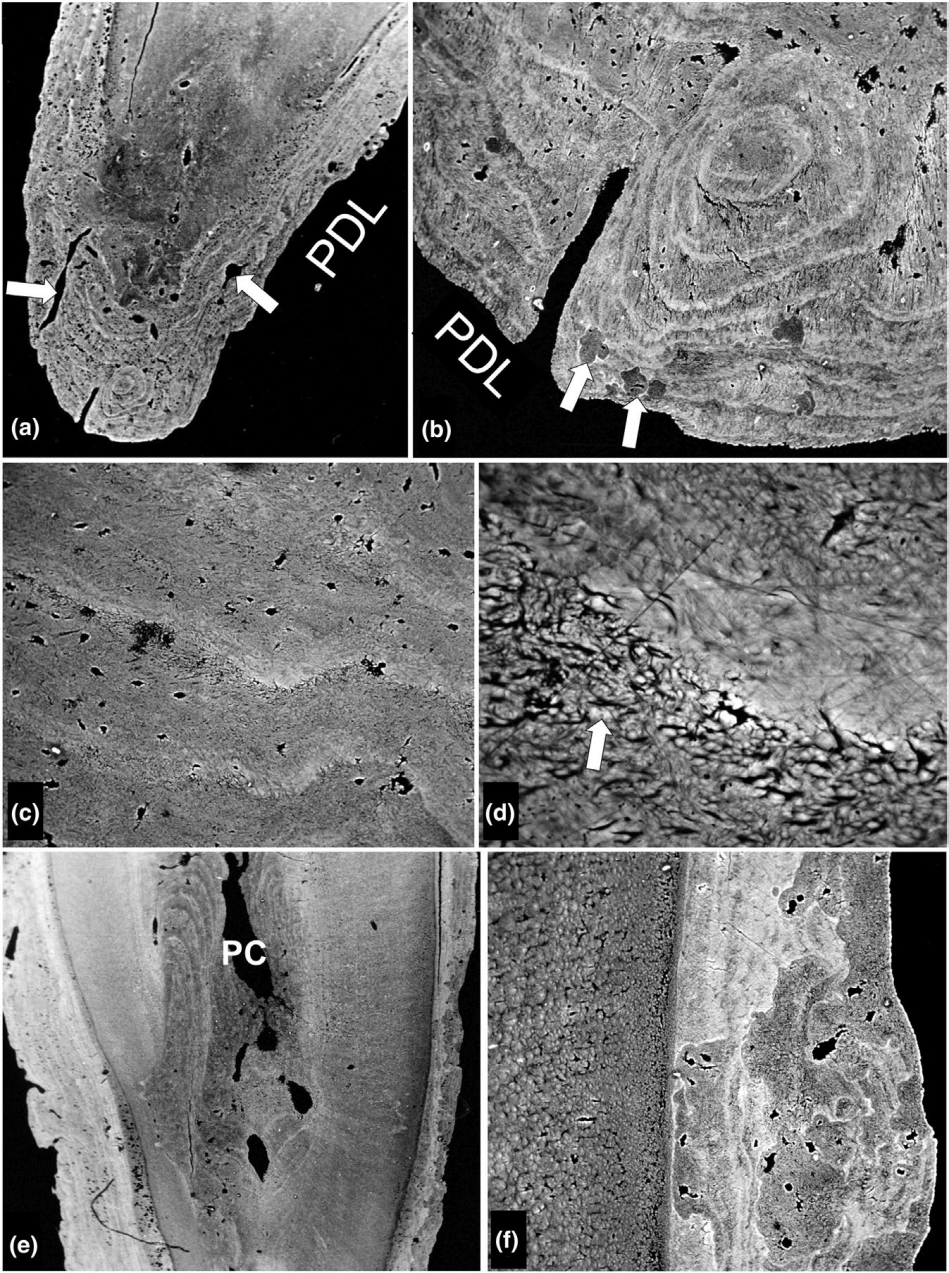
Nature of the cemental growth layers and ‘problems’ due to resorption. m1. (a) Distal root apex showing blood vessel canals (arrows) of the apical delta. FW 2575 μm. (b) Part of same apical delta of distal root of m1 showing several small resorption and repair patches (arrows), part of a single vascular canal connecting periodontal ligament (PDL) to pulp, and annual layers, some presenting as apparent circles due to the plane of section. General direction of growth in this field from top to bottom. FW 780 μm. (c) More detail showing nature of changes at growth arrest and recovery lines: Direction of growth in this field again from top to bottom of the image. Hypermineralised layers developed due to ‘maturation’ (an increase in mineral concentration after initial mineralisation) where growth stopped. Sharpey fibres seen in the transverse section are obscured by mineralisation in the arrest lines but can be distinguished in the newly deposited matrix. FW 290 μm. (d) Higher magnification showing how the space between the Sharpey fibres of the cementum (arrow) has filled in to create the dense resting line at the centre of the field of view. FW 83 μm. (e) Mid part of the mesial root. Multiple layers in cementum to the left [distal side] are mostly intact, while those at the right [mesial] side of the root have been largely remodelled. Multiple layers of atubular secondary dentine face the pulp chamber (PC). FW 3370 μm. (f) Mesial surface of the mesial root. A massive resorption patch has cut back through several annual layers which in turn had been re‐resorbed. This demonstrates active repair and would be called turnover in bone. FW 368 μm.

**FIGURE 3:**
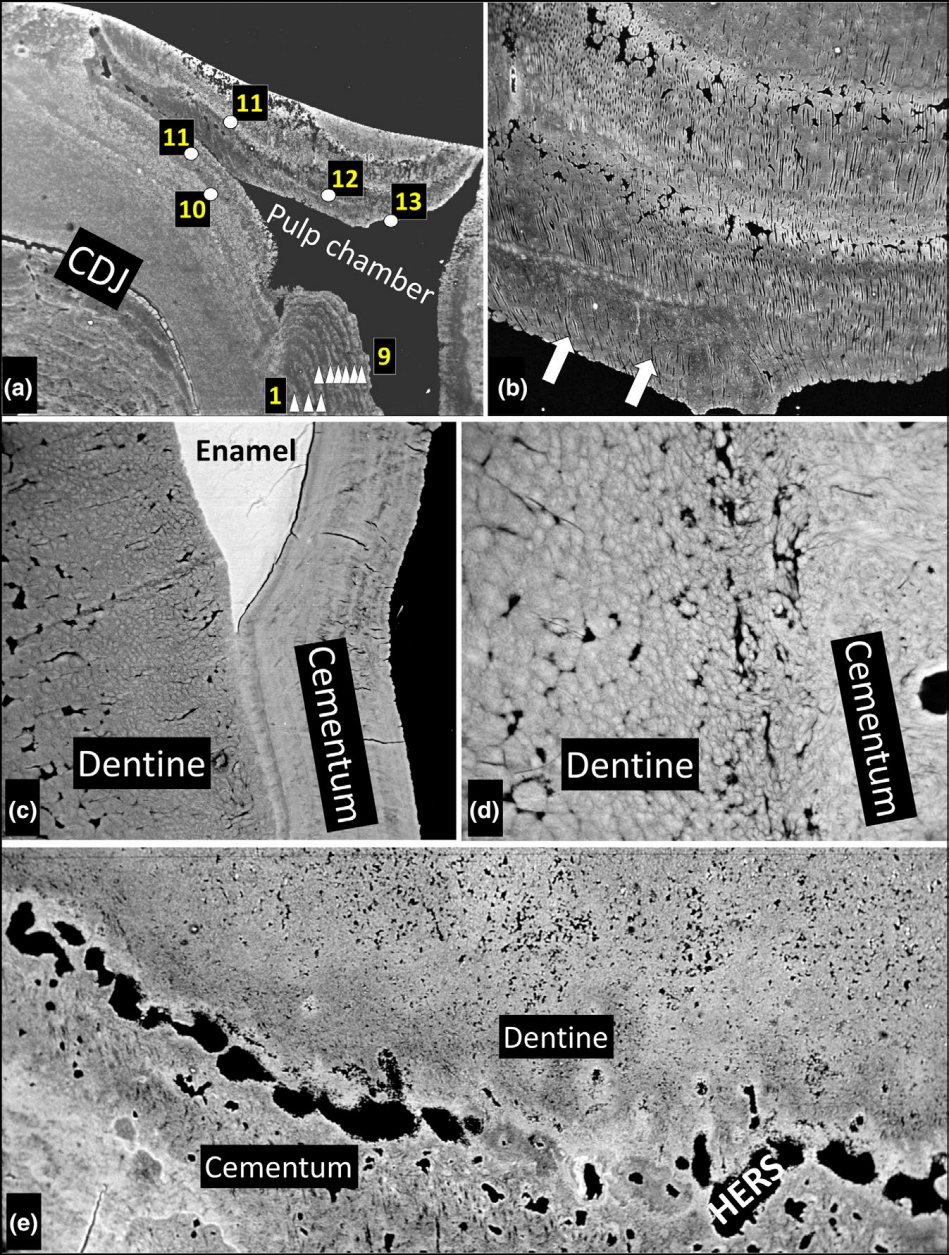
Growth layers in secondary dentine in m1, enamel‐cement junction, cement‐dentine junction and Hertwig's Epithelial Root Sheath (HERS). (a) Mesial pulp chamber of m1. The top of the image is the occlusal wear surface, deep to which are prominent layers which are taken to be annual increments of dentine formation. The layers can be traced round the top to the floor of the pulp chamber. The numbers indicate the winter of life in which each was formed. Cementum growth layers in the inter‐radicular ‘pad’ are seen in the bottom left‐hand corner of the field. FW 4055 μm. (b) m1. Pulp chamber roof, dentine growth layers, oblique longitudinal section of dentine tubules (arrows), Note two denser bands with prominent interglobular dentine. The mineralising front of the dentine, that is, the border between dentine and predentine, is at the bottom of the image. FW 830 μm. (c) Enamel‐cement‐dentine junctions in m2, showing that the thin growth layers in coronal cementum are continuous with those of the root cementum. FW 450 μm. (d) Mesial side of mesial root of m1 showing the intimate connection of dentine with cementum. FW 83 μm. (e) Dark voids at the dentine (top of the image) cement (bottom of the image) junction are spaces occupied by remnants of the Hertwig's epithelial root sheath (HERS). FW 760 μm.

Growth layers—annulations—showed as variations in the mineral content revealed by BSE‐SEM in cementum (Figures 1c–f, 2a–f and 3a). The bulk of cementum consists of broad, darker (i.e., less well mineralised) layers which correspond, respectively, to the ‘milky’, ‘brighter’, more light‐scattering bands seen by reflected light microscopy, the darker, more opaque, less translucent layers seen by transmitted light microscopy of undecalcified sections and the less densely stained (e.g., toluidine blue or haematoxylin) layers of decalcified sections. These are separated by narrow, whiter, more highly mineralised bands in which the Sharpey fibres are obscured because the intrinsic matrix between them is also better calcified (Figure 2a–d). We interpret these BSE‐SEM‐denser ‘seasonal annulations’ as the ‘winter’ or ‘rest’ lines which arise during slow or arrested matrix‐appositional growth. We counted 12 such lines, the first of which, labelled ‘1’ in Figure 1c, we propose was laid down during the first winter after birth during which the animal, born in June, would have been 0 whole years old. On this basis, she lived through 12 winters and died during her 13th, corresponding with the light layer outermost in the specimen (labelled ‘13’ in Figure 1c). This interpretation exactly matches the age determined by counting rest lines in a decalcified, stained section of i1 from the same individual.

There was evidence of substantial resorption and subsequent repair of cementum (Figures 1c and 2e,f). Such areas would of course have to be avoided during any layer‐counting procedure. Small resorption patches were also seen (Figure 2b).

Secondary dentine was also layered by mineral content. This was evident both through general differences in back‐scattering (i.e., signal intensity, grey level, mineral concentration) and through the number of non‐mineralised interglobular dentine (IGD—see Jones & Boyde, 1984) patches (Figure 3a,b). Secondary dentine was still forming in m1 at death: in the crown the dentine was mainly tubular with well‐marked IGD‐rich layers (Figure 3b), but in the lower pulp chamber and root it was largely without tubules.

In m2, in which enamel was present, the cemental layers were continuous between the tissue covering the crown and the root and there was therefore no difference between coronal and root surface dentine (Figure 3c). High‐magnification images showed that the union between cementum and dentine was so intimate as to obscure where one tissue ended and the other began (Figure 3d). Substantial non‐mineralised spaces were found at the cement‐dentine junction in the root apical regions in m2 (Figure 3e). We interpret these as inclusions of the Hertwig's Epithelial Root Sheath (HERS: or Epithelial Rests of Malassez, ERM) between the two tissues.

## 4 DISCUSSION

Determination of age at death is a central issue in animal ecology and counting annulations in teeth and other tissues is widely used for this purpose. Knowledge about the composition and process of formation of these structures, however, remains rudimentary. Here, we have shown how to create high‐resolution images of annulations in the teeth of a large wild mammal and have interpreted these in terms of the micro‐anatomical dynamics of dental tissue.

This study has shown that 20 kV BSE SEM images of flat surfaces of PMMA embedded blocks coated with a 20 nm layer of carbon to render them conductive—as depicted in Figures 1, 2, 3 in which ‘whiter’ means a higher mineral concentration—clearly demonstrate variation in mineral content in a very thin layer (depth 0.5 to 1 microns) in the surface of the sample. Since the lateral resolution is also less than 1 micron, the ‘voxel’ is much less than one cubic micron. It is important to keep in mind that the electron optical section that we employ is very thin: it means that we see exactly what is on the surface and do not average structures in depth as with optical and X‐ray methods.

Distinct growth layers in cementum, as in bone and dentine, may reflect variation in the fractions of (a) Type 1 collagen, (b) the ground substance protein‐polysaccharide complexes, (c) the water content of these components of the organic matrix and – what is observed in the present study – (d) the amount of this water which has been displaced by mineralisation with a carbonated hydroxyapatite.

Cementum shows thin densely mineralised annulations. This increase in mineral content would occur (in winter) when secretion of new matrix and the advance of the cementum mineralisation front slows or even ceases. This would be a passive process equivalent to the maturation (a rise in mineral concentration in already mineralised matrix) seen in mature, lamellar bone matrix and in the multiple tide marks of the advancing mineralisation front in articular calcified cartilage (Boyde, 2021).

The primary dentine in both teeth was a regular tubular dentine with some tubules partly, and some wholly, filled with peritubular dentine. The secondary dentine which filled the erstwhile pulp chamber contained clearly marked growth layers which bear some marked resemblance to those in the cementum. We have traced the matching layers in the two tissues in Figures 1c and 3a, from which we conclude that these lines are also annual.

The secondary dentine forms at all levels in the pulp chamber, that is, it is not just dentine which had been deposited as a physiological response to a stimulus caused by the exposure of tubules due to occlusal wear. In most instances this dentine appeared to contain no tubules, or at least they were difficult to discern because of the detail given by the innumerable small calcospherites in the BSE‐SEM images. New calcospherites are nucleated by the release of matrix vesicles from the odontoblasts (Jones & Boyde, 1984) and it follows that these cells continuously released matrix vesicles into the predentine matrix and therefore actively participate in ensuring that the dentine is properly mineralised.

The layers of secondary dentine formed below the worn occlusal surface were very thick which we interpret as their being part of a normal physiological response of the pulp to potential exposure. Extra layers would be deposited as required.

The exceptional non‐uniformity seen in the present study in the packing density of dentine tubules, in the amount of peritubular dentine and in the occurrence of non‐tubular regions is remarkable and has not, to our knowledge, been commented on in the literature.

The inclusion of epithelial cell remnants of Hertwig's epithelial root sheath (HERS) or the epithelial rests of Malassez (ERM) in the tooth between dentine and cementum has been clearly documented in murine molars (Boyde & Bromage, 2021; Lester & Boyde, 1970). To our knowledge, this has not been previously described in large teeth in long‐lived mammals.

## 5 CONCLUSIONS

The layering of the cementum which is the basis of age‐determination, is consonant with the proposal that the rate at which new tissue is deposited varies with season. The rate slows in winter, coinciding with the spontaneous seasonal entry into a catabolic phase characteristic of *Rangifer* and other northern cervids (Tyler et al., 2020). Then the last formed tissue increases its mineral content in a process analogous with maturation in bone and articular calcified cartilage. In secondary (Haversian system) osteons, the maturation stage increase in mineral content starts at the last formed, free, internal surface. Likewise, in articular calcified cartilage, the advance of the mineralising front which converts hyaline articular cartilage to articular calcified cartilage is periodically arrested: the mineral content increases at the temporary arrest line and spreads ‘backwards’ into the less well mineralised, deeper tissue (Boyde, 2021).

Counter to the conclusions of Lieberman (1994), we found (a) that cementum surfaces are frequently resorbed in a patch‐wise fashion, probably to repair injuries sustained when the periodontal ligament is compacted by abnormal occlusal forces (e.g., biting on a particle of stone) such that alveolar bone comes into contact with cement, or at least the periodontal ligament is crushed and (b) that dentine formation persists throughout life.

## AUTHOR CONTRIBUTIONS

NJCT collected the material, AB prepared the material, performed the BSE‐SEM imaging and digitised and analysed the images and prepared Figures for publication. Both authors contributed to conceptualisation and writing and reviewing and editing successive versions of the manuscript. Both authors gave final approval to the submitted version.

## CONFLICT OF INTEREST STATEMENT

The authors have no conflict of interest.

## Data Availability

Data are available from the authors on reasonable request.
